# Numerical Study on Elastic Properties of Natural Fibres in Multi-Hybrid Composites

**DOI:** 10.3390/polym17223031

**Published:** 2025-11-15

**Authors:** Mughees Shahid, Gediminas Monastyreckis, Daiva Zeleniakiene

**Affiliations:** Department of Mechanical Engineering, Kaunas University of Technology, Studentu Str. 56, LT-51424 Kaunas, Lithuania; mughees.shahid@ktu.edu (M.S.);

**Keywords:** sustainable composites, hybrid composites, finite element analysis, elastic properties

## Abstract

This study investigates the elastic properties of bio-epoxy composites reinforced with natural fibres (flax, hemp) and synthetic fibres (S-glass), with particular focus on the effect of the fibre volume fraction (VF) ranging from 10% to 70%. Three-dimensional representative volume element (RVE) models were developed for single-fibre, hybrid, and multi-fibre systems. The mean-field homogenisation (MF) approach, based on the Mori–Tanaka scheme, and finite element analysis (FEA) with periodic boundary conditions were employed to predict the effective elastic properties, including longitudinal, transverse, and shear moduli, as well as Poisson’s ratio. These numerical predictions were validated against analytical models, including the rule of mixtures, Chamis, and composite cylinder assemblage (CCA) methods. The results demonstrate that increasing the VF enhances longitudinal, transverse, and shear moduli while reducing Poisson’s ratio in natural fibre composites. The good agreement between numerical, semi-analytical, and analytical methods validates the 3D RVE models as useful tools for predicting the properties of multi-hybrid natural fibre composites, supporting their design for lightweight structural applications.

## 1. Introduction

Natural fibre composites (NFCs) have emerged as sustainable alternatives to conventional synthetic composites in various engineering applications, driven by their cost-effectiveness, satisfactory mechanical performance, and reduced environmental impact [1,2,3]. These materials are increasingly employed in sectors such as construction, aerospace, automotive, and marine engineering for semi-structural and secondary components, due to their favourable specific properties and bio-based origin [4]. The global market for NFCs has experienced steady growth, reflecting both increasing industrial adoption and the widespread availability of suitable regional fibres. From 2016 to 2024, the sustainable composites market demonstrated a compound annual growth rate (CAGR) of 11.8% [5]. Among various natural fibres, flax and hemp have attracted significant attention for structural use in the past decade, owing to their promising mechanical properties and compatibility with polymer matrices [6].

Flax fibre stands out among natural fibres for its superior mechanical characteristics [7], attributable to its high cellulose content (60–70 wt%), significant crystallinity (50–90%), low microfibrillar angle (<10°), and high aspect ratio [8]. These structural features contribute to its exceptional tensile strength, which arises from its elongated primary fibres and highly aligned microfibrillar orientation. The Young’s modulus of elementary flax fibres varies with diameter, ranging from 39 to 78 GPa for fibres from 35 to 5 μm [7,9,10], with variations often linked to differences in fibre lumen size. These favourable properties have spurred growing international research interest in flax fibres and their composites [11,12,13]. Niphaphun et al. [14] reported a flax fibre modulus of 19.4 GPa, using a fibre area correction method. They found close agreement between experimental and predicted results for unidirectional composites (13.2 GPa at 25% fibre volume fraction (VF)), confirming the modelling accuracy for natural fibre composites. In a separate study on flax yarns, Abida et al. [15] observed that Young’s modulus, diameter, and tensile strength follow Gaussian distributions. Their numerical approach for assessing yarn quality through fabric tensile tests yielded a Young’s modulus of 9.4 ± 0.9 GPa, slightly lower than the experimental value of 10.8 ± 1.3 GPa. Further expanding applications, Sofie et al. [16] investigated straw flax as reinforcement in polylactic acid composites, demonstrating a 62% improvement in longitudinal stiffness and a 10% increase in specific stiffness compared to short glass fibre composites. This highlights the potential of flax-based composites for developing lightweight, high-performance, sustainable materials.

Hemp is a bast fibre derived from the plant stem and is cultivated primarily in Europe and Asia [17,18]. It is recognised for its notable mechanical strength [19] and hierarchical structure, in which cellulose microfibrils are embedded within a matrix of lignin and hemicellulose. This microstructure makes it particularly suitable as a reinforcement in lightweight composite materials [20]. Peyman et al. [21] investigated the relationship between bundle diameter and tensile properties of hemp fibres, noting that Young’s modulus increases with increasing bundle diameter. Benjamin et al. [22] quantified uncertainties in the elastic properties of woven hemp fabric-reinforced GreenPoxy (bio-based thermoset epoxy resin) composites, highlighting the challenges in predicting behaviour, due to natural variability. Sheedev et al. [23] investigated thermoplastic composites reinforced with woven hemp and found that elevated temperatures caused polymer softening and fibre degradation, which in turn reduced the overall mechanical performance. In a related study, Laura et al. [24] demonstrated that increasing the VF of twill hemp fabric reinforcement enhances tensile, flexural, and impact properties in thermoplastic composites, confirming hemp’s suitability for structural use.

Several researchers have proposed hybridising NFCs with synthetic fibres such as basalt, carbon, and glass [25,26,27,28] to enhance their mechanical performance. Hybridisation in composites refers to combining two or more different fibre types within a single matrix to form a hybrid composite. Interfacial interactions between the matrix and reinforcements affect composite performance. The type and quality of these interactions can increase or decrease tensile strength, impact resistance, and toughness [29]. In composite materials, morphological differences correspond to variations in the physical structure, geometry, and arrangement of the fibres and the matrix. The main aspects of morphological differences are the fibre orientation, VF, fibre length, phase continuity, fibre distribution, and packing. Understanding and controlling morphology enables increased composite performance in lightweight structural applications, such as the aerospace and automotive sectors [30]. These factors contribute to the distinctive behaviour of hybrid composites, enabling their performance to be tailored to the intended application. Srinivasan et al. [27] evaluated hybrid composites incorporating flax and banana fibres, reporting a significant improvement in flexural strength, with an ultimate stress of 13.54 N/mm^2^ compared to 9.76 N/mm^2^ for single-fibre banana composites. This hybrid system also exhibited superior impact resistance, absorbing 16 J of energy versus 12 J for pure flax composites. Chandra et al. [31] developed a hybrid system combining hemp/flax fibres with waste glass dust as particulate fillers. These studies underscore the viability of hybrid natural-synthetic composites in enhancing both mechanical and functional characteristics.

In recent years, computational techniques have become increasingly important for simulating mechanical properties and facilitating the design of NFCs [32,33]. Consequently, numerical and analytical models are now extensively employed in NFC design and fibre modelling. Commonly used analytical micromechanical models include the Mori–Tanaka scheme [34], Halpin–Tsai approach [35], Cox model [36], Voigt–Reuss [37], modified Rule of Mixtures (ROM) [38], Composite Cylinder Assemblage (CCA) [39], mechanics of materials approach [40], method of cells [41], and the effective medium theory [33,42]. For microscale analysis, representative volume element (RVE) modelling has proven particularly effective for simulating composite properties [43,44], with numerous methodologies available for predicting various NFC characteristics [45,46]. Most research has focused on predicting elastic properties, including Young’s modulus, shear modulus, and Poisson’s ratio, with studies demonstrating acceptable agreement between model predictions and experimental results [47,48]. For instance, Niphaphun et al. [14] confirmed the accuracy of analytical methods in predicting flax fibre composite performance, while Sowmya et al. [49] validated finite element analysis (FEA) results for hemp nanofibre composites. Mohamad et al. [47] investigated the effect of fibre volume fraction on the elastic properties of palm/luffa NFCs, using several analytical methods (Chamis, Halpin–Tsai, Nielsen, and ROM) to validate the FEA results. Similarly, Ayyappa et al. [50] demonstrated through numerical and experimental analyses that hybrid composites exhibit improved mechanical properties compared with single-fibre systems, particularly highlighting the influence of the matrix material on composite performance.

The current literature review indicates that research on natural fibre composites has predominantly focused on single-fibre systems (e.g., banana, flax yarn, woven twill hemp fabric, short glass fibre) and hybrid systems (e.g., flax/banana, palm/luffa, hemp/flax with glass waste). The existing research indicates limited work on the elastic properties of a multi-hybrid fibre system combined with S-glass fibre over a wide range of VF (0.1–−0.7), highlighting a research gap for further investigation. Furthermore, the application of computational modelling approaches, such as 3D RVE analysis, for multi-hybrid fibre systems has not been fully explored. Thus, there is a need to investigate these fibre systems to examine the elastic properties of single (flax/epoxy, hemp/epoxy, and glass/epoxy), hybrid (FH/epoxy, FG/epoxy, and HG/epoxy), and multi-fibre systems (FHG/epoxy) by developing a 3D RVE to make them suitable for lightweight structural applications.

This research aims to develop and analyse a 3D micromechanical RVE model to predict the elastic properties of bio-epoxy composites reinforced with natural fibres (flax, hemp) and synthetic fibres (S-glass) over a wide range of VF (0.1–0.7). For this purpose, three theoretical approaches were employed—numerical (FEA), semi-analytical (Mori–Tanaka homogenisation scheme), and analytical (ROM, Chamis, and CCA)—to predict the elastic properties, including the longitudinal, transverse, and shear moduli, as well as Poisson’s ratio. In addition, the computed numerical results were validated against semi-analytical and analytical predictions. The findings provide valuable insights into how different fibre systems and the VF influence the elastic properties of polymer composites, thereby supporting the development of eco-friendly, lightweight composites for structural applications.

## 2. Methodology

**Figure 1:**
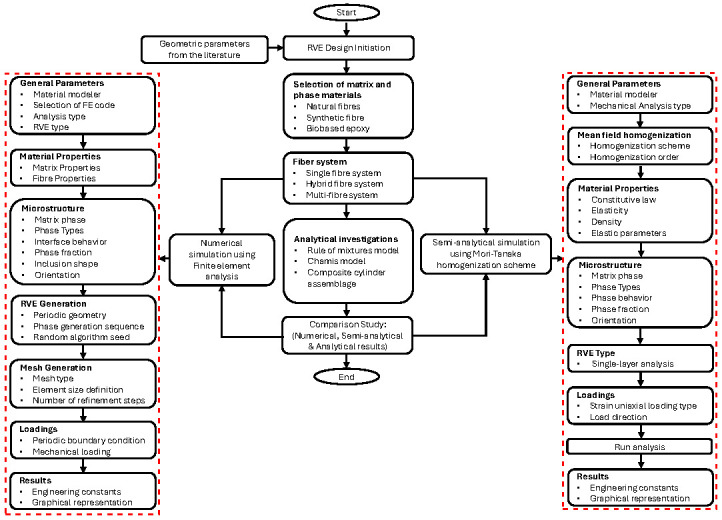
Flowchart of the research and numerical methodology.

The bio-epoxy (termed as epoxy) matrix was reinforced with flax, hemp, and S-glass (termed as glass) fibres. All constituent materials were modelled as isotropic [47,50] and were assumed to form a homogeneous, void-free composite structure, free from fibre waviness and moisture sensitivity. A uniform fibre distribution was adopted to minimise interfacial gaps and ensure consistent fibre–matrix interactions [51,52]. This assumption idealises the composite for a baseline comparison. Natural fibres exhibit anisotropy and lumen voids; therefore, the predicted stiffness represents an upper-bound response under perfect bonding and uniform fibre distribution [53,54]. For hybrid and multi-fibre systems, fibres were incorporated in equal proportions. The overall research methodology is summarised in Figure 1.

### 2.1. Finite Element Analysis

**Figure 2:**
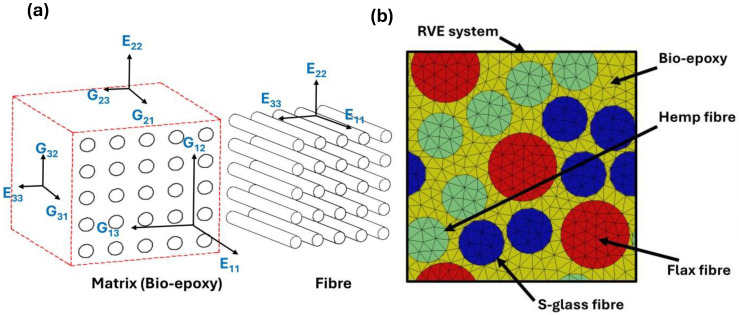
Composite structure and RVE for FEA. (**a**) Tensor notation of FRC and (**b**) homogenised RVE.

**Figure 3:**
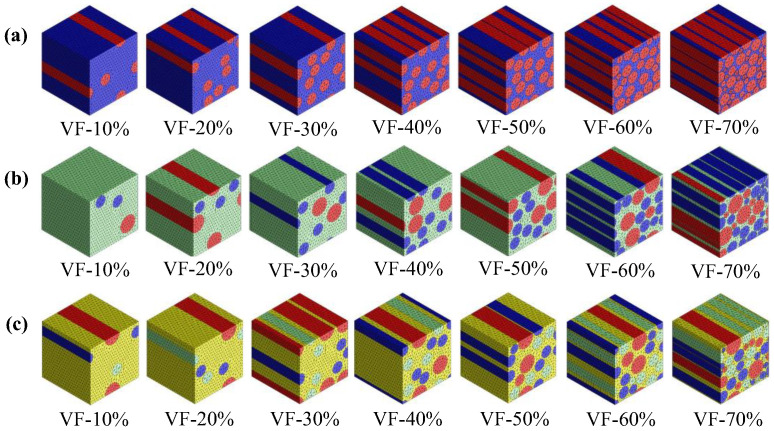
FEA 3D meshed models by VF (%). (**a**) Single, (**b**) hybrid, and (**c**) multi-fibre system.

The elastic properties of the NFCs were determined through FEA using Digimat 2017 [46,55,56,57]. A square RVE (100 µm × 100 µm × 100 µm) was developed with fibre diameters of 20 μm for hemp and glass, and 30 μm for flax fibres [58,59,60]. These values were chosen to model typical fibres used in composite materials, and to ensure that model results agree with the experimental results reported in previous studies [12,61,62,63]. The three-dimensional constitutive relationship for anisotropic materials, implemented in the FEA, is defined by equations (S1, S2) [51,64,65]. In this study, the unidirectional FRCs are treated as transversely isotropic, since they exhibit isotropic behaviour in the plane that is perpendicular to the fibre direction. When the stiffness tensor C is ascertained, the effective elastic characteristics are computed in equations (S3–S6) [51]. The composite microstructure and RVE system used in the simulations are shown in Figure 2, where Figure 2a illustrates the tensor notation applied to a fibre-reinforced composite (FRC), including fibre and matrix, and Figure 2b shows the homogenised RVE as having different fibres, such as flax, hemp, and glass, within the epoxy matrix. All composites were modelled without voids, assuming perfect matrix–fibre adhesion. The material properties used as inputs for these simulations are provided in Table 1. The fibre properties were obtained from the literature and the manufacturer’s datasheet, whereas the bio-epoxy matrix modulus was experimentally determined through a laboratory test [66,67,68,69,70,71,72,73,74,75,76,77,78,79,80,81,82,83]. To evaluate the reliability of the predicted results, a simple parametric sensitivity analysis was performed at 0.5 VF, using the same micromechanical formulations (numerical, semi-analytical, and analytical). The fibre modulus was varied by ±10% (52.80 GPa and 64.50 GPa), which caused a nearly proportional change by ±10% (26.97 GPa and 32.83 GPa) in the predicted longitudinal modulus (E_1_). The transverse modulus (E_2_), shear modulus (G_23_), and Poisson’s ratio (ν_12_) were less affected because they are matrix-dominated properties. The stiffness trend among flax, hemp, and S-glass-based systems remained unchanged, confirming that the comparative trends are insensitive to reasonable variations in the input parameters. Figure 3 presents the 3D meshed models for different fibre systems from (10% to 70%) VF. The main reason behind choosing this range is to provide a comprehensive understanding of the micromechanical behaviour of natural and hybrid bio-epoxy composites. To ensure adequate matrix impregnation and processability, bio-composites often use lower VF values: typically 0.1–0.4 [30]. In comparison, composites with a higher VF (0.5–0.7) are representative of structural-grade composites, which are usually used in lightweight engineering applications, such as car panels and semi-structural elements [84]. Figure 3a shows the single-fibre system with uniform fibre distribution. Figure 3b illustrates the hybrid fibre system containing two fibre types, offering an improved strength-to-weight ratio compared to single-fibre systems. Figure 3c displays the multi-fibre system incorporating three fibre types (flax, hemp, and glass), which enhances stress distribution and mechanical performance. The multi-fibre system improves elastic properties by enabling a more compact fibre arrangement at higher VFs. Conformal meshing with a tetrahedral element was applied to all RVE models, followed by the implementation of periodic boundary conditions to ensure an accurate prediction of effective elastic properties.

### 2.2. Mesh Convergence Study

**Figure 4:**
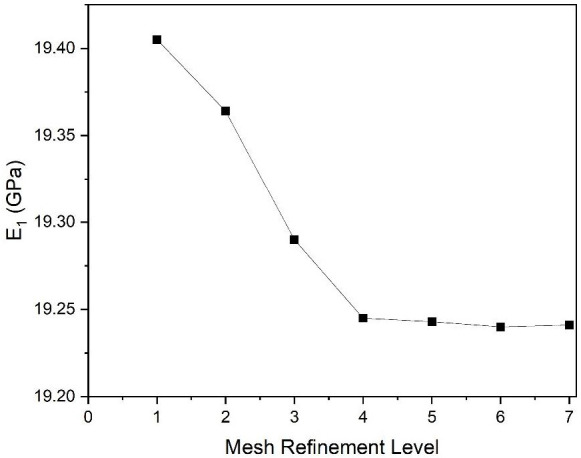
Variation in E_1_ with different mesh refinement levels. The square markers indicate the computed E_1_ values at each refinement level, and the solid line illustrates the overall trend.

A mesh convergence study was conducted to ensure the accuracy and reliability of numerical simulations. The number of elements and nodes directly influences both the precision of the results and the computational cost. Additionally, solutions were evaluated across multiple mesh refinement levels until stability was achieved. E_1_ was computed at various refinement levels to establish mesh convergence, as shown in Figure 4. The optimal mesh configuration (refinement level 5) consisted of 82,321 elements and 134,031 nodes, with an average skewness of 0.812. The mesh independence study was performed using 3D tetrahedral elements on the single-fibre flax/epoxy RVE. The same mesh configuration and refinement level 5 were subsequently applied to all fibre systems, including hybrid and multi-fibre composites. The relative error between refinement levels 5 and 6 was 0.005, and between levels 5 and 7 it was 0.010, confirming that mesh convergence was achieved at refinement level 5 for all configurations. As demonstrated in Figure 4, results exhibited negligible variation beyond this refinement level, confirming mesh convergence. This mesh configuration provides an optimal balance between computational accuracy and efficiency for the current study. In addition, the numerical solver’s convergence criterion was set to a relative residual tolerance of 1 × 10^−4^, ensuring numerical stability and accuracy. Detailed mesh statistics for all refinement levels are provided in Table 2.

### 2.3. Mean Field Homogenisation

The semi-analytical simulations in this study employed the Mori–Tanaka homogenisation scheme, also referred to as mean-field homogenisation (MF). This method predicts the effective elastic properties of composite materials by analysing their microstructural characteristics, making it particularly suitable for materials with a complex internal structure [8,18]. Within this framework, the Mori–Tanaka approach was applied to determine the effective elastic properties of various fibre systems. An RVE was constructed with an epoxy matrix containing a VF between 10% and 70%. Both the fibres and epoxy matrix were treated as isotropic materials, with their properties provided in Table 1. The simulations were conducted using Digimat-MF 2017 [55,57], which implements the Mori–Tanaka method based on Eshelby’s inclusion theory. According to this theoretical framework, the matrix is modelled as a homogeneous elastic medium, while the fibres are treated as ellipsoidal inclusions. The Eshelby tensor establishes the relationship between the inclusion’s strain and its eigenstrains, providing the fundamental mechanical basis for the homogenisation scheme. For fibrous reinforcements, the Eshelby tensor takes a specific form that is appropriate for spheroidal inclusions, which underpins the mathematical formulation of the Mori–Tanaka method as implemented in Digimat-MF, in equations (S7–S16) [85].

### 2.4. Analytical Methods

The current study incorporates analytical approaches to validate the results of FEA and MF, including the Chamis, ROM, and CCA models. These models use various mathematical formulae to assess the elastic properties of composites and NFCs.

#### 2.4.1. Rule of Mixture

ROM is usually used to determine the composite elastic properties by utilising matrix and fibre parameters, fibre orientations, and the VF. The elastic properties were described by equations (S17–S20).

Note: In the following equations, E_L_ and E_T_ denote the longitudinal and transverse moduli of elasticity, respectively, which are equivalent to E_1_ and E_2_ in the manuscript.

#### 2.4.2. Chamis Model

The Chamis model is an extensively used semi-empirical model, developed from ROM and incorporating the square root of the VF [86]. The equations (S21–S24) were used to predict the elastic properties.

#### 2.4.3. Composite Cylinder Assemblage

The composites serve as periodic RVEs in elasticity models called composite cylinder assembly (CCA) models. The CCA model makes the following presumptions: a continuous fibre is surrounded by a cylindrical matrix, the fibres are placed in a periodic structural sequence, and they have a circular cross-section [87]. The elastic properties were calculated using equations (S25–S36).

## 3. Results and Discussions

### 3.1. Longitudinal Modulus

**Figure 5:**
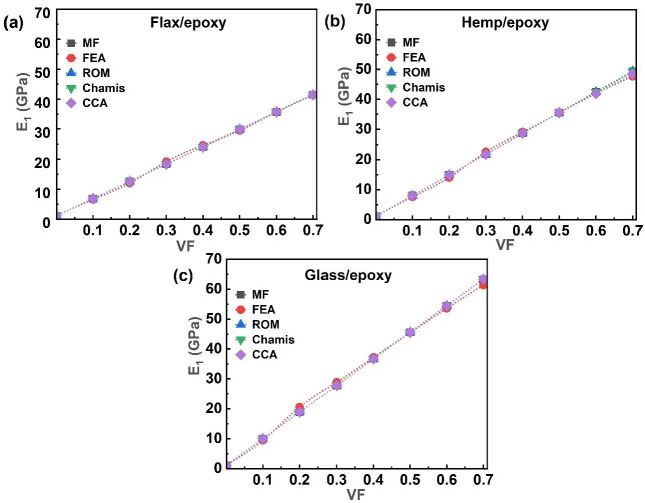
Single-fibre system E_1_ of (**a**) flax/epoxy, (**b**) hemp/epoxy, and (**c**) glass/epoxy.

**Figure 6:**
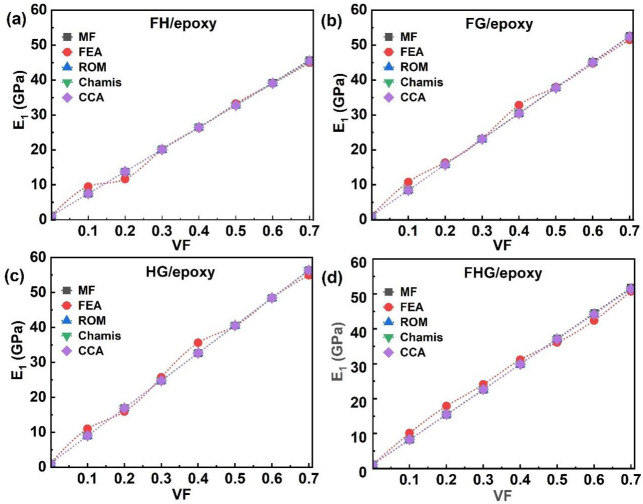
Hybrid and multi-fibre systems E_1_ of (**a**) FH/epoxy, (**b**) FG/epoxy, (**c**) HG/epoxy, and (**d**) FHG/epoxy.

The longitudinal modulus (E_1_), defined as the ratio of longitudinal stress to longitudinal strain, characterises the stiffness of a composite under axial loading. Figure 5 presents the E_1_ values for flax/epoxy, hemp/epoxy, and glass/epoxy composites, as predicted by analytical, MF, and FEA models. As shown in Figure 5a–c, the results from the FEA and MF models agree closely with those from the analytical methods across all single-fibre systems. A consistent increase in the E_1_ with the increasing VF was observed across all composites. Specifically, in the FEA, the E_1_ of flax/epoxy composites increased from 6.58 GPa to 41.42 GPa, as VF rose from 0.1 to 0.7 (Figure 5a). Similarly, the E_1_ of hemp/epoxy composites increased from 7.59 GPa to 47.69 GPa over the same VF range (Figure 5b). In contrast, glass/epoxy composites exhibited a more gradual increase in E_1_, from 9.48 GPa at 0.1 VF to 61.38 GPa at 0.7 VF (Figure 5c). For instance, in MF, at 0.1 VF, flax/epoxy and hemp/epoxy composites showed E_1_ values of 6.89 GPa and 8.02 GPa, respectively, in NFCs. In comparison, glass/epoxy reached 63.34 GPa at 0.7 VF in a synthetic fibre composite, the highest E_1_ value among all single-fibre systems. Minor discrepancies were observed between FEA results and those from MF and analytical models. As the VF increases in composite materials, the E_1_ improves because the fibres, being stronger and stiffer than the matrix, bear a greater portion of the load. With a higher VF, the efficiency of load transfer between the fibres and the matrix increases, thereby enhancing the composite’s resistance to deformation. Figure 6 further extends this analysis to hybrid and multi-fibre systems, including flax–hemp (FH)/epoxy, flax–glass (FG)/epoxy, hemp–glass (HG)/epoxy, and flax–hemp–glass (FHG)/epoxy composites, illustrating how fibre hybridisation influences longitudinal stiffness.

For the hybrid fibre systems, the E_1_ values for FH/epoxy, FG/epoxy, and HG/epoxy, obtained from analytical models, showed good agreement with both the MF and FEA predictions. As described in Figure 6a, in the FEA, the E_1_ of FH/epoxy increased from 9.51 GPa to 44.93 GPa as the VF rose from 0.1 to 0.7. In the case of FG/epoxy, in Figure 6b, the E_1_ increased from 10.77 GPa to 51.48 GPa with 0.7 VF. Similarly, the E_1_ of HG/epoxy rose consistently from 11 GPa to 54.95 GPa (0.1 to 0.7) VF (Figure 6c). The main reason for this behaviour is variation in their material properties and fibre diameters. Stronger fibres, such as glass, enhance overall stiffness and strength, while natural fibres like flax and hemp offer sustainability and cost benefits. The highest E_1_ value among hybrid systems, 56.35 GPa, was achieved by the HG/epoxy by using the MF model at 0.7 VF. Overall, the FEA results agreed with the analytical and MF models for the FH/epoxy and HG/epoxy systems, whereas FG/epoxy showed progressive alignment at higher VFs.

As shown in Figure 6d, the E_1_ of the multi-fibre FHG/epoxy composite increased with the VF across all approaches, including analytical, MF, and FEA. The MF model yielded the highest E_1_ value of 51.72 GPa at 0.7 VF. The incorporation of glass fibres into natural fibre systems resulted in a notable enhancement in stiffness, with the HG/epoxy system reaching a maximum E_1_ of 56.35 GPa and the glass/epoxy system achieving the highest overall value of 63.34 GPa at 0.7 VF, using MF homogenisation. The main reason for this behaviour is the higher load-carrying capacity of the fibres compared to the epoxy matrix. With the increasing VF, more fibres, such as flax, hemp, and glass, share the applied stress, thereby enhancing the composite’s longitudinal stiffness and strength. These results align with the trends observed in Figure 5 and Figure 6, confirming that increasing the VF consistently improves the E_1_, due to the greater load-bearing capacity of stiffer fibres [13,24,88]. At 0.4 VF, FEA predicted an elastic modulus of 24.67 GPa for flax/bio-epoxy. This agrees well with the experimental values reported as 22.8 GPa [61] and 23.9 ± 1.9 GPa [62] at 0.4 VF. Similarly, at 0.5 VF, FEA yielded 29.44 GPa, which is consistent with the experimental results of 26 ± 2 GPa at 0.5 VF [12] and 31.4 GPa at 0.51 VF [63]. This confirms that the numerical predictions and experimental measurements agree at 0.4 and 0.5 VFs.

### 3.2. Transverse Modulus

**Figure 7:**
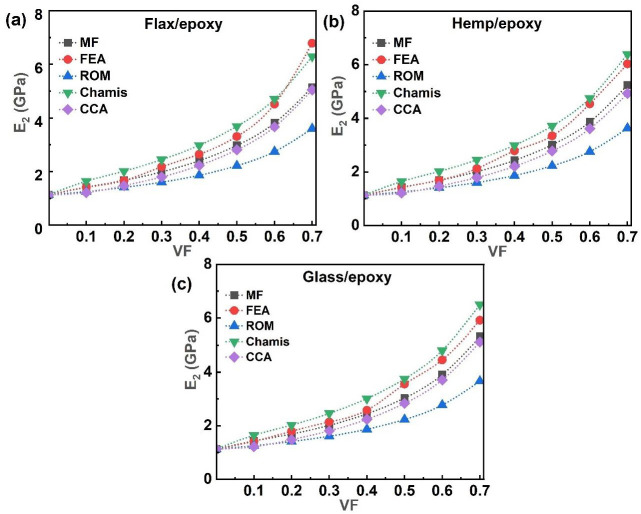
Single fibre system E_2_ of (**a**) flax/epoxy, (**b**) hemp/epoxy, and (**c**) glass/epoxy.

**Figure 8:**
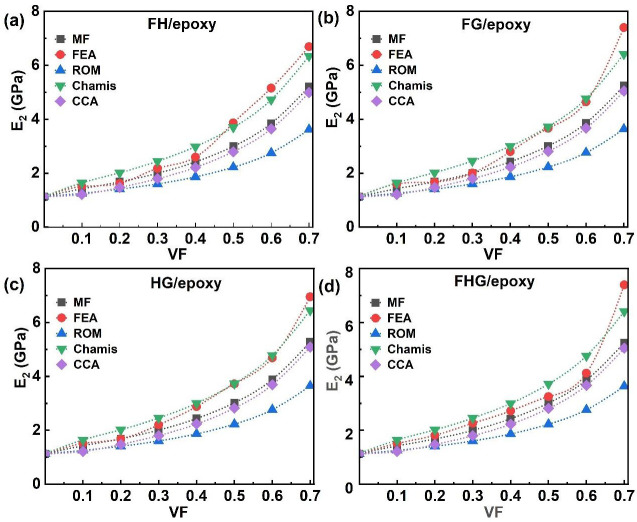
Hybrid and multi-fibre systems E_2_ of (**a**) FH/epoxy, (**b**) FG/epoxy, (**c**) HG/epoxy, and (**d**) FHG/epoxy.

The transverse modulus (E_2_) is an important property governing the behaviour of fibre-reinforced epoxy composites, as it directly influences stiffness, load-bearing capacity, and resistance to deformation perpendicular to the fibre direction. Figure 7 presents the evolution of E_2_ as a function of the VF (0.1 to 0.7) for single-fibre systems reinforced with flax, hemp, and glass fibres. The graphs in Figure 7a–c show that the E_2_ increased with the VF across the MF, FEA, and analytical models, all of which exhibited a similar trend in E_2_ behaviour. ROM yielded the lowest predictions for E_2_, while the Chamis model provided the highest values. For instance, at 0.1 VF of flax fibres, the CCA model predicted a minimum E_2_ of 1.20 GPa. In contrast, at 0.7 VF, the maximum predicted E_2_ values were 6.79 GPa for flax/epoxy using FEA, 6.50 GPa for glass/epoxy, and 6.39 GPa for hemp/epoxy, as obtained from the Chamis model. Figure 8 further extends this analysis to hybrid and multi-fibre systems (FH/epoxy, FG/epoxy, HG/epoxy, and FHG/epoxy), showing their E_2_ behaviour across the same VF range (0.1–0.7).

As shown in Figure 8a, the E_2_ of FH/epoxy increased with the VF across all modelling methods (MF, FEA, and analytical), with the CCA model predicting the lowest values (1.20–1.22 GPa) at 0.1 VF. In contrast, FEA predicted the highest E_2_ value of 7.40 GPa at 0.7 VF for FG/epoxy composites, as the incorporation of glass fibres alongside flax enhanced the transverse stiffness, relative to other composites (Figure 8b). Conversely, the HG/epoxy system showed a reduced E_2_ of 6.95 GPa at 0.7 VF (Figure 8c), with a further decrease to 6.69 GPa for the FH/epoxy composite.

As illustrated in Figure 8d, the E_2_ values for the multi-fibre FHG/epoxy system increased with the VF across all models. The FEA model predicted the highest value, 7.39 GPa, at 0.7 VF, whereas ROM yielded the lowest (3.64 GPa). Overall, FG/epoxy achieved the highest E_2_ values, while the introduction of hemp fibres with flax fibres generally reduced transverse stiffness. These trends align well with MF and CCA predictions with all systems. The differences in the nominal values are due to the assumptions of each method. The rapid increase in the E_2_ with the VF assumes perfect fibre–matrix adhesion; however, in real laminates, partial debonding could limit the E_2_, meaning the predicted values represent upper bounds [8,84,89,90]. Numerical FEA provides more accurate and reliable results, whereas analytical and semi-analytical models are valuable for quick estimation and trend prediction, especially during the early design stage. The consistent increase in E_2_ with VF [66,91] highlights the influence of fibre content in enhancing transverse properties, although the matrix characteristics remain the dominant factor controlling transverse deformation behaviour.

### 3.3. Shear Modulus

**Figure 9:**
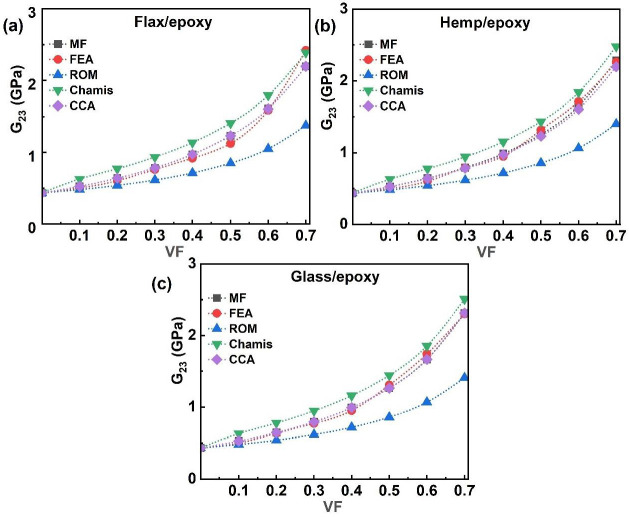
Single-fibre system G_23_ of (**a**) flax/epoxy, (**b**) hemp/epoxy, and (**c**) glass/epoxy.

The in-plane shear modulus (G_23_), defined as the ratio of shear stress to shear strain, reflects a material’s resistance to deformation under shear loading. Figure 9 presents the G_23_ values for single-fibre systems (flax/epoxy, hemp/epoxy, and glass/epoxy), predicted using FEA, MF, and analytical models. All models consistently showed an increase in the G_23_ as the VF increased. As depicted in Figure 9a–c, the Chamis model yielded the highest predictions, while the ROM gave the lowest. At 10% VF, the G_23_ values ranged between 0.48 and 0.63 GPa across all composites. Maximum values reached 2.51 GPa for glass/epoxy, 2.48 GPa for hemp/epoxy, and 2.42 GPa for flax/epoxy composites at 0.7 VF. The predictions from the FEA, MF, and CCA models were correlated closely across all single-fibre systems.

**Figure 10:**
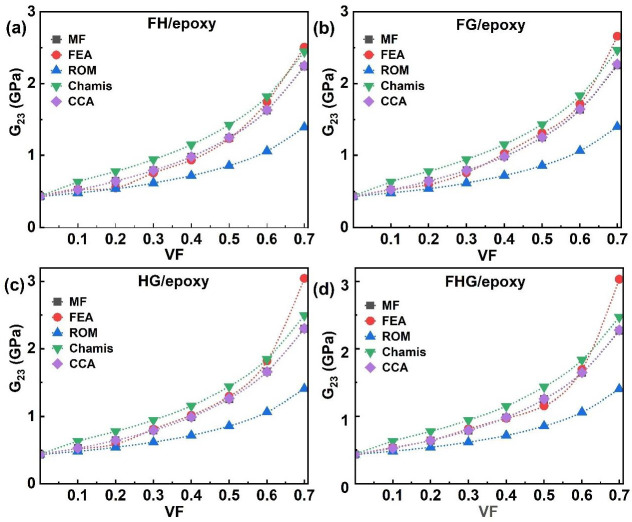
Hybrid and multi-fibre systems G_23_ of (**a**) FH/epoxy, (**b**) FG/epoxy, (**c**) HG/epoxy, and (**d**) FHG/epoxy.

The G_23_ values for hybrid and multi-fibre systems (FH/epoxy, FG/epoxy, HG/epoxy, and FHG/epoxy) across different VF values (0.1–0.7) are presented in Figure 10. The MF, FEA, and analytical models consistently demonstrated that the G_23_ increases with the rising VF, a trend also observed for FH/epoxy (Figure 10a), FG/epoxy (Figure 10b), and HG/epoxy (Figure 10c) composites. The highest G_23_ value of 3.05 GPa was observed for HG/epoxy at 0.7 VF using the FEA model, while ROM gave the lowest value of 1.40 GPa for FH/epoxy at the same VF. FG/epoxy and FH/epoxy attained peak values of 2.66 GPa and 2.51 GPa, respectively. MF and CCA models showed good agreement in predicting G_23_ for most hybrid systems, though FEA results began diverging beyond 0.6 VF for FH/epoxy and 0.5 VF for FG and HG composites. Overall, the G_23_ increased steadily with the fibre content, demonstrating close agreement among model predictions except at a higher VF, where minor deviations were observed between the FEA, MF, and CCA results.

For the multi-fibre FHG/epoxy system, all models showed a consistent increase in the G_23_ with the rising VF, as illustrated in Figure 10d. The FEA, Chamis, CCA, and MF models produced nearly identical predictions. The FEA model yielded the highest G_23_ value of 3.03 GPa at 0.7 VF, whereas ROM predicted the lowest value of 1.40 GPa at the same VF. The variations in the nominal values arise from the assumptions made by each method. At a higher 0.6 VF, fibre–fibre proximity and local stress concentration cause numerical divergence, whereas analytical models assume uniform strain; this explains the slight offset [58]. This difference arises from physical microstructural effects, rather than numerical instability. The incorporation of glass fibres contributed to a moderate increase in shear stiffness, attributed to their enhanced resistance to interfacial shear deformation. In contrast, a higher flax content resulted in reduced G_23_ values, consistent with the matrix-dominated nature of shear properties in natural fibre composites [66,92,93]. These trends align with the behaviour observed in both single-fibre and hybrid systems (Figure 9 and Figure 10), reinforcing the critical role of fibre type and content in shaping the shear performance of multi-phase composites.

### 3.4. Poisson’s Ratio

**Figure 11:**
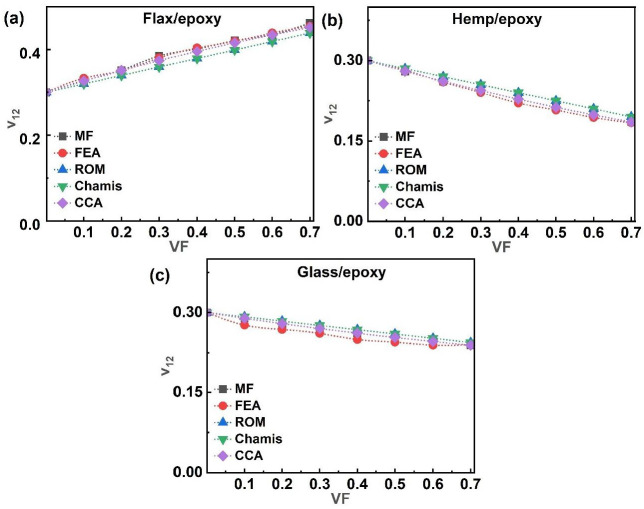
Single-fibre system ν_12_ of (**a**) flax/epoxy, (**b**) hemp/epoxy, and (**c**) glass/epoxy.

Poisson’s ratio (ν_12_) characterises the transverse contraction of a material under longitudinal tensile strain. Figure 11 shows the ν_12_ values for glass/epoxy, flax/epoxy, and hemp/epoxy, as predicted by analytical, MF, and FEA models. In Figure 11a–c, the analytical results indicate distinct trends: the ν_12_ decreased with the increasing VF for hemp/epoxy and glass/epoxy composites but increased for the flax/epoxy systems as the VF increased. These trends were consistently observed across all modelling approaches. At 70% VF, the ν_12_ for flax/epoxy rose from 0.32 at 0.1 VF to 0.46 at 0.7 VF, while hemp/epoxy was 0.28 at 0.1 VF, and declined to 0.19 at a VF of 0.70. Glass/epoxy showed intermediate behaviour, with the ν_12_ declining from 0.29 to 0.24 over the same VF range. The FEA results showed acceptable agreement with both analytical and MF predictions for all systems.

**Figure 12:**
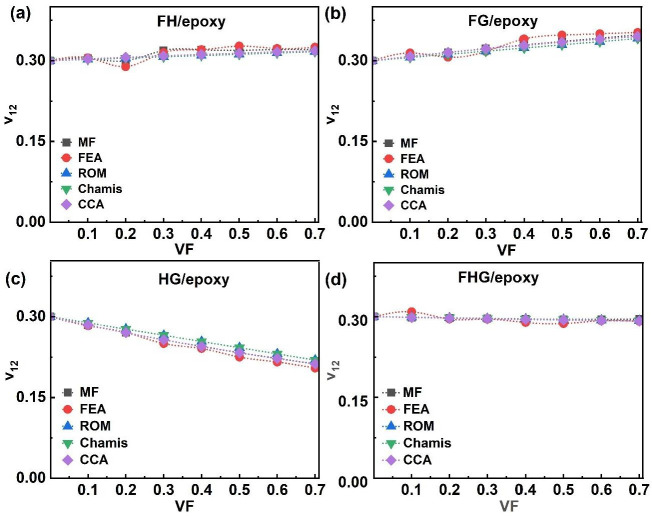
Hybrid and multi-fibre systems ν_12_ of (**a**) FH/epoxy, (**b**) FG/epoxy, (**c**) HG/epoxy, and (**d**) FHG/epoxy.

Figure 12 presents the ν_12_ of hybrid and multi-fibre systems (FH/epoxy, FG/epoxy, HG/epoxy, and FHG/epoxy). In the hybrid fibre systems (FH/epoxy, FG/epoxy, and HG/epoxy), the ν_12_ values obtained from analytical models agreed with those derived from the MF and FEA methods. Minor deviations in the FEA results were observed at certain VF values: notably, at 0.2 and 0.5 VF for FH/epoxy, and at 0.4 VF for FG/epoxy. As illustrated in Figure 12a, the ν_12_ for FH/epoxy increased marginally from 0.30 to 0.32 as VF rose from 0.1 to 0.7. A similar increasing trend was noted for FG/epoxy (Figure 12b), where ν_12_ rose from 0.31 to 0.35 at 0.7 VF. In contrast, ν_12_ for HG/epoxy decreased from 0.29 to 0.20 over the same VF range (Figure 12c).

For the multi-fibre FHG/epoxy system (Figure 12d), the ν_12_ showed a consistent decrease with the increasing VF across all models. In FHG/epoxy (Figure 12d), the ν_12_ decreased from 0.31 at 0.1 VF to 0.29 at 0.7 VF. Overall, the analytical, MF, and FEA predictions remained consistent. Flax fibres possess larger lumens and lower transverse stiffness, allowing greater lateral deformation of the surrounding matrix [11,53]. In contrast, hemp and glass fibres are denser and stiffer, constraining the transverse strain and resulting in lower ν_12_ values [54]. Finally, the ν_12_ generally decreased with the VF for all systems except those containing flax (flax/epoxy, FH/epoxy, and FG/epoxy), where it increased [11]. This inverse relationship between the ν_12_ and the elasticity in flax composites suggests that Poisson’s ratio reflects not only the elastic response but also potential variations in deformability, which is consistent with earlier findings [94].

### 3.5. Composite Density

**Figure 13:**
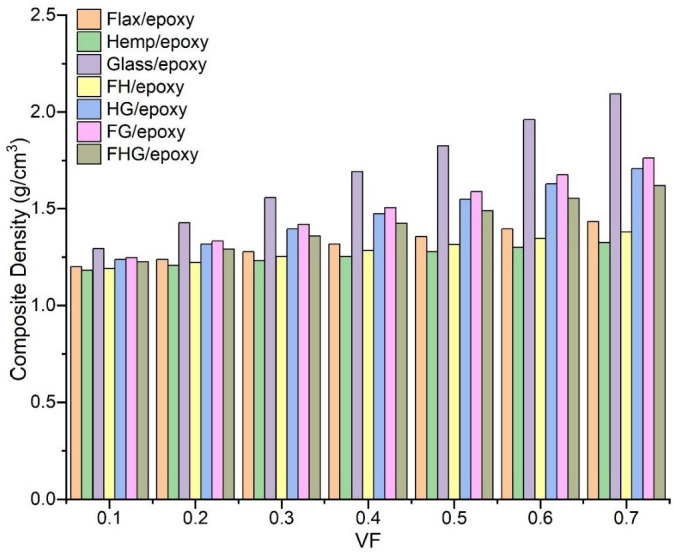
Comparative investigation of single, hybrid, and multi-fibre systems’ composite densities for (0.10 to 0.70) VF.

The density of a polymer composite is a fundamental property that significantly influences its overall mechanical performance and application potential [95]. The composite’s density can be effectively controlled by adjusting the fibre type and VF. Composite densities were predicted using the rule-of-mixtures approach, based on constituent densities, as described by Equation (S37) [95]. As shown in Figure 13, density increases consistently with the VF across all fibre systems [67]. It is evident that, in the case of FH/epoxy, the composite density decreased compared to the single fibres of flax and hemp. This indicates that the combination of these natural fibres results in a more efficient packing structure. In contrast, introducing glass fibres into natural fibre systems increased composite density, as observed in FG/epoxy and HG/epoxy composites [96]. The multi-fibre FHG/epoxy system exhibited an optimised density distribution, balancing the lightweight properties of natural fibres against the performance advantages provided by glass reinforcement. This optimisation highlights the potential of hybrid composites to achieve enhanced stiffness and strength while maintaining a low overall weight.

### 3.6. Comparative Study

**Figure 14:**
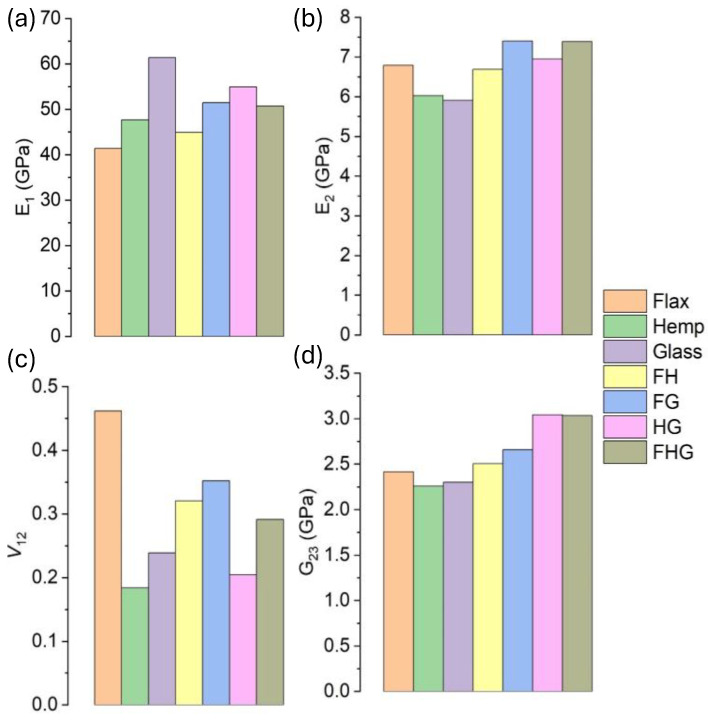
The comparative study of elastic properties at 0.7 VF: (**a**) E_1_ of single, hybrid, and multi-fibre systems; (**b**) E_2_ of single, hybrid, and multi-fibre systems; (**c**) Poisson’s ratio ν_12_; (**d**) Shear modulus G_23_.

This comparative study evaluates the performance of hybrid and multi-fibre systems, relative to single-fibre systems. The comparative elastic properties of different composite systems at 0.7 VF are illustrated in the bar graphs in Figure 14. For the FH/epoxy hybrid system, the E_1_ increased by 8.48% compared to flax/epoxy but decreased by 5.80% relative to hemp/epoxy (Figure 14a). As shown in Figure 14b, the E_2_ decreased by up to 1.42% for flax/epoxy and 74.97% for hemp/epoxy. In the case of the ν_12_, it improved for the flax/epoxy composite, while it showed a significant decrease of 75.06% for hemp/epoxy (Figure 14c). Meanwhile, in Figure 14d, the G_23_ increased by 3.68% in flax/epoxy and 11.04% in hemp/epoxy for both single fibre systems.

For the FG/epoxy hybrid system, the E_1_ showed a 24.29% increase compared to flax/epoxy, but a 16.13% decrease compared to glass/epoxy (Figure 14a). The E_2_ values rose by 9.01% and 25.14% for flax/epoxy and glass/epoxy, respectively (Figure 14b). The ν_12_ value decreased by 23.73% in the flax/epoxy composite but increased by 47.35% in the glass/epoxy composite (Figure 14c). For G_23_ in Figure 14d, the value also improved, rising by 9.96% in flax/epoxy and 15.54% in glass/epoxy. In the case of the HG/epoxy hybrid system, in Figure 14a, the E_1_ increased by 14.56% compared to hemp/epoxy but dropped by 7.92% compared to glass/epoxy. In Figure 14b, the E_2_ values improved in both cases, with increases of 18.18% and 22.77% for hemp/epoxy and glass/epoxy, respectively. In Figure 14c, the ν_12_ value fell by 10.84% in hemp/epoxy but rose by 14.18% in glass/epoxy. Similarly, in Figure 14d, the G_23_ improved in both cases, with increases of 20.55% and 25.82% for hemp/epoxy and glass/epoxy, respectively.

Finally, as illustrated in Figure 14a, the FHG/epoxy multi-fibre system exhibited a 23.17% increase in the E_1_, relative to flax/epoxy, and a 4.37% increase compared with hemp/epoxy. However, it showed a 16.11% reduction compared to glass/epoxy. For E_2_ in Figure 14b, all three single-fibre systems showed improvements: 5.82% for flax/epoxy, 9.73% for hemp/epoxy, and 13.99% for glass/epoxy. In Figure 14c, the ν_12_ value increased by 35.71% in flax/epoxy but decreased by 57.29% and 21.79% for hemp/epoxy and glass/epoxy, respectively. As shown in Figure 14d, the G_23_ increased across all three systems by 14.26% for flax/epoxy, 10.33% for hemp/epoxy, and 15.15% for glass/epoxy.

As shown in Table 3, the anisotropy ratio for the flax/epoxy composite is 6.10. The anisotropy ratio represents the stiffness of the composite along the fibre direction compared to the transverse direction. The equation (S38) was used to calculate the composite’s anisotropy ratio. This value indicates how much stiffer the material is in the axial direction. A ratio of 6.10 for flax/epoxy indicates that the composite is moderately anisotropic, with balanced stiffness behaviour. In contrast, the glass/epoxy composite exhibits an anisotropy ratio of 10.38, suggesting that it is very stiff along the fibre direction, but significantly weaker transverse to it. The hybrid systems (ranging from 6.7 to 7.9) exhibit intermediate anisotropy, suggesting that combining fibres reduces directional differences and provides a more uniform stiffness. In the case of reinforcement efficiency, this parameter indicates how effectively the fibres contribute to the composite’s overall stiffness. The equation (S39) was used to determine the composite’s reinforcement efficiency. A value of 1.0 represents an excellent fibre–matrix load transfer, corresponding to nearly ideal bonding conditions. All systems show values between 0.97 and 1.0, indicating almost perfect reinforcement efficiency. This means the composites exhibit strong interfacial bonding, as expected under the assumed modelling conditions. Therefore, these results confirm that the numerical models used are physically realistic and the predicted composite stiffness values are accurate.

Furthermore, the hybrid effect index (HEI) represents the synergy arising when different fibres are combined, which can be either positive or negative. A positive HEI indicates that the hybrid performs better than the simple average of its constituent single-fibre composites. The HEI was computed by equation (S40). From the table, the FH/epoxy shows a small positive effect of +0.84%, indicating a slight hybrid improvement. The FG/epoxy combination exhibits an HEI of +0.15%, reflecting almost linear behaviour with neither loss nor gain in stiffness. The HG/epoxy shows a more substantial effect of +0.76%, and FHG/epoxy exhibits the highest value of +1.05%, demonstrating a pronounced synergy among all fibres. In summary, a positive HEI indicates that hybridisation enhances composite stiffness beyond the simple rule of mixtures, which is particularly beneficial for lightweight, weight-sensitive structural applications. Physically, the HEI quantifies the synergistic stiffness gain arising from fibre–fibre interactions and improved stress-transfer efficiency within the hybrid composite. Specific stiffness is a measure of a material’s lightweight efficiency, defined as the ratio of its modulus to the density, and it is expressed by equation (S41). From the table, it is evident that the hemp/epoxy composite exhibits the highest specific stiffness (36.05 GPa·cm^3^·g^−1^), indicating superior stiffness-to-weight performance. The hybrid composites exhibit balanced stiffness values of 31–32 GPa·cm^3^·g^−1^, whereas flax/epoxy and glass/epoxy composites show similar values of approximately 29 GPa·cm^3^·g^−1^. This high specific stiffness makes the materials particularly suitable for lightweight structural applications, especially in automotive and aerospace sectors, where achieving both strength and weight reduction is essential.

A comparative study of single, hybrid, and multi-fibre systems using numerical (FEA), semi-analytical (Mori–Tanaka), and analytical (ROM, Chamis, and CCA) approaches at 0.7 VF (including error percentages) is presented in Table 4. For single-fibre systems, the differences between FEA, semi-analytical and analytical predictions are negligible, with deviations below 0.1% for flax/epoxy and below 3.5% for hemp/epoxy and glass/epoxy. For hybrid systems, including FH/epoxy, FG/epoxy, HG/epoxy, and FHG/epoxy, the deviation remains below 2%, except for HG/epoxy, which is slightly above 2.5%. This indicates that the models accurately capture the hybrid stiffness behaviour. The results show good agreement among all methods. The maximum deviation was less than 3.5%, confirming the reliability of the proposed micromechanical framework. No fitting parameters were adjusted, and identical input data were used across FEA, MF, and analytical models, confirming the independence of the validation.

Overall, hybrid and multi-fibre systems generally enhance the elastic properties compared with single-fibre composites, although the extent of improvement depends on the fibre combination. FH/epoxy exhibited moderate gains in the E_1_ and G_23_, but showed reductions in the E_2_ and ν_12_, relative to hemp composites. FG/epoxy and HG/epoxy systems exhibited significant increases in both E_1_ and E_2_, confirming the reinforcing effect of glass fibres when combined with natural fibres. The multi-fibre FHG/epoxy system achieved balanced improvements across most properties, particularly in the E_1_, E_2_, and G_23_, thereby highlighting the synergistic effect of combining multiple fibres. These findings suggest that careful fibre selection and hybridisation can strategically optimise the stiffness, shear response, and deformation characteristics of polymer composites.

## 4. Conclusions

This study developed and analysed 3D micromechanical representative volume element (RVE) models to predict the elastic properties of bio-epoxy composites reinforced with natural fibres (flax and hemp) and synthetic fibres (S-glass). The investigation focused on single, hybrid, and multi-fibre systems across a wide range of fibre volume fractions (VF), from 0.1 to 0.7. Key findings from the numerical (finite element analysis), semi-analytical (Mori–Tanaka mean-field homogenisation), and analytical (rule of mixtures, Chamis, and composite cylinder assemblage) models are summarised as follows:

1. A consistent increase in the longitudinal modulus (E_1_) was observed with the increasing VF for all systems. Hybrid and multi-fibre systems generally exhibited higher stiffness than single natural fibre composites. The S-glass/epoxy system achieved the highest E_1_ value (63.34 GPa at 0.7 VF). In contrast, the multi-fibre flax–hemp–glass (FHG)/epoxy system demonstrated a balanced improvement, providing a 23.17% increase over flax/epoxy.
2. Both the transverse modulus (E_2_) and shear modulus (G_23_) increased with the VF across all systems. The incorporation of glass fibres, particularly in the flax–glass (FG)/epoxy hybrid system, resulted in the highest transverse and shear stiffness. These results emphasise the matrix–dominated nature of these properties while highlighting the significant reinforcing effect of a higher fibre content.
3. The behaviour of Poisson’s ratio (ν_12_) was highly dependent on fibre type. Composites containing flax fibre showed an increase in the ν_12_ with the VF, whereas systems with hemp or glass fibres exhibited a decrease. This inverse relationship in flax composites suggests a complex interaction between fibre properties and composite deformability.
4. The density of the composites increased with the VF. Hybridising natural fibres (flax–hemp) resulted in a more efficient packing structure and a lower density than the individual fibres. The introduction of glass fibres increased the composite density and enhanced the elastic modulus, while the multi-fibre FHG system provided an optimised balance between weight and performance.
5. Good agreement was obtained between the finite element analysis (FEA), mean-field homogenisation (MF), and analytical models, confirming the accuracy of the computational approaches adopted. The mesh convergence study further verified the reliability of the FEA results.

In conclusion, this research demonstrates that hybridisation and multi-fibre reinforcement are effective strategies for enhancing the elastic properties of natural fibre composites. Hybridisation reduces dependence on synthetic glass fibres and lowers the overall cost while improving specific stiffness. As incorporating several fibre types increases the lay-up complexity, the design must balance performance with manufacturability. The combination of natural fibres with synthetic glass fibres significantly improved stiffness while allowing for density optimisation. The application of 3D RVE modelling provides a computational framework for the future design and development of high-performance, sustainable composite materials for structural applications.

## Figures and Tables

**Table 1:** The input properties for the investigated matrices and reinforcements.

Materials	Density (g/cm^3^)	Young’s Modulus (GPa)	Poisson’s Ratio	Shear Modulus (GPa)
Flax	1.55 [66,67]	58.6 [66,67,68]	0.50 [69]	19.6
Hemp	1.393 [70]	70.0 [71,72]	0.15 [73,74]	30.4
S-glass	2.495 [75,76,77]	93.0 [78,79,80]	0.23 [81]	36.9
Bio-epoxy	1.159 [82] *	1.13 **	0.30 [82,83]	0.435

* Properties from the manufacturer, ** lab experiment.

**Table 2:** Details of mesh convergence study.

Mesh Refinement Level	Number of Elements	Number of Nodes	E_1_ (GPa)	Relative Error
1	19,407	33,967	19.405	0.845
2	29,704	50,736	19.364	0.635
3	54,062	89,328	19.290	0.254
4	67,312	110,262	19.245	0.021
5	82,321	134,031	19.243	0.010
6	103,669	166,234	19.240	0.005
7	173,873	273,837	19.241	

**Table 3:** Comparative study of single, hybrid, and multi-fibre systems with anisotropic ratio, reinforcement efficiency, hybrid effect index, and specific stiffness at 0.7 VF.

Fibre System	Anisotropy Ratio (A_E_)	Reinforcement Efficiency (ηE_1_, %)	Hybrid Effect Index (HEI)	Specific Stiffness (E_1_/ρ) (GPa·cm^3^·g^−1^)
Flax/epoxy	6.10	1.0	-	28.91
Hemp/epoxy	7.91	0.97	-	36.05
Glass/epoxy	10.38	0.97	-	29.31
FH/epoxy	6.71	0.99	0.84	32.61
FG/epoxy	6.96	0.98	0.15	29.19
HG/epoxy	7.91	0.98	0.76	32.16
FHG/epoxy	6.86	0.99	1.05	31.26

**Table 4:** Comparative study of single, hybrid, and multi-fibre systems with numerical, semi-analytical, and analytical results at 0.7 VF.

Fibre System	Numerical	Semi-Analytical		Analytical					
	E1	E1	Error	E1 (ROM, GPa)	Error (ROM, %)	E1 (Chamis, GPa)	Error (Chamis, %)	E1 (CCA, GPa)	Error (CCA, %)
	(FEA, GPa)	(MF, GPa)	(MF, %)						
Flax/epoxy	41.42	41.4	0.05	41.39	0.07	41.39	0.07	41.4	0.05
Hemp/epoxy	47.69	49.35	3.46	49.34	3.45	49.34	3.45	48.41	1.5
Glass/epoxy	61.38	63.34	3.2	63.34	3.2	63.34	3.2	63.34	3.2
FH/epoxy	44.93	45.69	1.69	45.36	0.97	45.36	0.97	45.39	1.03
FG/epoxy	51.48	52.57	2.13	52.36	1.72	52.36	1.72	52.37	1.73
HG/epoxy	54.95	56.35	2.56	56.34	2.53	56.34	2.53	56.35	2.54
FHG/epoxy	50.69	51.72	2.03	51.36	1.32	51.36	1.32	51.36	1.32

## Data Availability

The original contributions presented in this study are included in the article/Supplementary Material. Further inquiries can be directed to the corresponding author.

## Supplementary Materials

The following supporting information can be downloaded at: https://www.mdpi.com/article/10.3390/polym17223031/s1. The document includes detailed formulations for FEA, MF, ROM, Chamis, CCA models, and all equations used in the study.
