# Supplementary material for: Numerical Study on Elastic Properties of Natural Fibres in Multi-Hybrid Composites
Source: Polymers (Basel). 2025 Nov 15;17(22):3031. doi: 10.3390/polym17223031 (PMC12656229; doi:10.3390/polym17223031)
Supplement: Supplementary file 1 [file polymers-17-03031-s001.zip › polymers-3933273-supplementary.pdf]

Supporting Information

Numerical Study on Elastic Properties of Natural Fibres in Multi-Hybrid Composites

Finite Element Analysis

$$\begin{Bmatrix} \sigma_1 \\ \sigma_2 \\ \sigma_3 \\ \sigma_4 \\ \sigma_5 \\ \sigma_6 \end{Bmatrix} = \begin{pmatrix} C_{11} & C_{12} & C_{13} & C_{14} & C_{15} & C_{16} \\ C_{12} & C_{22} & C_{23} & C_{24} & C_{25} & C_{26} \\ C_{13} & C_{23} & C_{33} & C_{34} & C_{35} & C_{36} \\ C_{14} & C_{24} & C_{34} & C_{44} & C_{45} & C_{46} \\ C_{15} & C_{25} & C_{35} & C_{14} & C_{55} & C_{16} \\ C_{16} & C_{26} & C_{36} & C_{45} & C_{55} & C_{66} \end{pmatrix} \begin{Bmatrix} \varepsilon_1 \\ \varepsilon_2 \\ \varepsilon_3 \\ \varepsilon_4 \\ \varepsilon_5 \\ \varepsilon_6 \end{Bmatrix} \quad \text{(S1)}$$

$$\begin{Bmatrix} \sigma_1 \\ \sigma_2 \\ \sigma_3 \\ \sigma_4 \\ \sigma_5 \\ \sigma_6 \end{Bmatrix} = \begin{pmatrix} C_{11} & C_{12} & C_{13} & 0 & 0 & 0 \\ C_{12} & C_{22} & C_{23} & 0 & 0 & 0 \\ C_{13} & C_{23} & C_{33} & 0 & 0 & C_{36} \\ 0 & 0 & 0 & \left(\dfrac{C_{22}-C_{23}}{2}\right) & 0 & 0 \\ 0 & 0 & 0 & 0 & C_{66} & 0 \\ 0 & 0 & 0 & 0 & 0 & 0 \end{pmatrix} \begin{Bmatrix} \varepsilon_1 \\ \varepsilon_2 \\ \varepsilon_3 \\ \varepsilon_4 \\ \varepsilon_5 \\ \varepsilon_6 \end{Bmatrix} \quad \text{(S2)}$$

$$E_L = C_{11} - \frac{2C_{12}^{\ 2}}{C_{22}+C_{23}}$$

(S3)

$$E_T = C_{11} - \frac{2C_{12}^{\ 2}}{C_{22}+C_{23}} \frac{\left[C_{11}\left(C_{22}+C_{23}\right)-2C_{12}^{\ 2}\right]}{\left(C_{11}C_{22}-C_{12}^{\ 2}\right)}\left(C_{22}-C_{23}\right) \quad \text{(S4)}$$

$$v_{in-plane} = \frac{C_{12}}{C_{22}+C_{23}} \quad \text{(S5)}$$

$$G_T = C_{66} \quad \text{(S6)}$$

Where,

σ : Stress vector

ε : Strain vector

C : Material stiffness matrix

E_L : Longitudinal modulus of elasticity

E_T : Transverse modulus of elasticity

$\upsilon_{in\text{-}plane}$: In-plane Poisson's ratio

G_T : Transverse shear modulus

Mean Field Homogenisation

$$e^c_{ij} = \mathrm{S}_{ijkl}\, e^*_{kl} \tag{S7}$$

$$S_{ijkl} = S_{jikl} = S_{ijlk} \tag{S8}$$

$$S_{jikl} \neq S_{ijlk} \tag{S9}$$

$$S_{1111} = \frac{1}{2(1-v_o)}\left\{1-2v_o+\frac{3\alpha^2-1}{\alpha^2-1}-\left[1-2v_o+\frac{3\alpha^2}{\alpha^2-1}\right]g\right\} \tag{S10}$$

$$S_{2222} = S_{3333} = \frac{3}{8(1-v_o)}\left(\frac{\alpha^2}{\alpha^2-1}\right)+\frac{1}{4(1-v_o)}\left[1-2v_o-\frac{9}{4(\alpha^2-1)}\right]g \tag{S11}$$

$$S_{2233} = S_{3322} = \frac{1}{4(1-v_o)}\left\{\frac{\alpha^2}{2(\alpha^2-1)}-\left[1-2v_o+\frac{3}{4(\alpha^2-1)}\right]g\right\} \tag{S12}$$

$$S_{2211} = S_{3311} = -\frac{1}{2(1-v_o)}\left(\frac{\alpha^2}{\alpha^2-1}\right)+\frac{1}{4(1-v_o)}\left\{\frac{3\alpha^2}{\alpha^2-1}-(1-2v_o)\right\}g \tag{S13}$$

$$S_{1122} = S_{1133} = -\frac{1}{2(1-v_o)}\left[1-2v_o+\frac{1}{\alpha^2-1}\right]+\frac{1}{2(1-v_o)}\left[1-2v_o+\frac{3}{2(\alpha^2-1)}\right]g \tag{S14}$$

$$S_{2323} = S_{3232} = \frac{1}{4(1-v_o)}\left\{\frac{\alpha^2}{2(\alpha^2-1)}+\left[1-2v_o-\frac{3}{4(\alpha^2-1)}\right]g\right\} \tag{S15}$$

$$S_{1212} = S_{1313} = \frac{1}{4(1-v_o)}\left\{1-2v_o-\frac{\alpha^2+1}{\alpha^2-1}-\frac{1}{2}\left[1-2v_o-\frac{3(\alpha^2+1)}{\alpha^2-1}\right]g\right\} \tag{S16}$$

Where,

e : Strain tensor

S : Stiffness tensor

v_o : Poisson’s ratio of the material

g : Scaling factor

α: Aspect ratio of the fibre length to the fibre diameter

Rule of Mixture

$$E_L = E_f V_f + E_m V_m \tag{S17}$$

$$v_{in-plane} = v_f V_f + v_m V_m \tag{S18}$$

$$E_T = \frac{E_m E_f}{E_m V_f + E_f V_m} \tag{S19}$$

$$G_T = \frac{G_m G_f}{G_m V_f + G_f V_m} \tag{S20}$$

Where,

E_f: Modulus of elasticity of the fibre

E_m : Modulus of elasticity of the matrix

V_f : Volume fraction of the fibre in the composite

V_m : Volume fraction of the matrix in the composite

Chamis Model

$$E_L = E_f V_f + E_m V_m \tag{S21}$$

$$v_{in-plane} = v_f V_f + v_m V_m \tag{S22}$$

$$E_T = \frac{E_m}{1 - \left\{ \sqrt{V_f} \left[1 - \left(\frac{E_m}{E_f} \right) \right] \right\}} \tag{S23}$$

$$G_T = \frac{G_m}{1 - \left\{ \sqrt{V_f} \left[1 - \left(\frac{G_m}{G_f} \right) \right] \right\}} \tag{S24}$$

Where,

G_m : Shear modulus of the matrix material

G_f : Shear modulus of the fiber material

Composite Cylinder Assemblage

$$E_L = E_f V_f + E_m V_m - \frac{2E_m\, E_f V_f \left(v_f - v_m\right)^2 \left(1 - V_f\right)}{E_f B_1 + E_m B_2} \tag{S25}$$

$$v_{in-plane} = v_f V_f + v_m V_m + \frac{\left(V_f V_m\ \left(v_f - v_m\right)\left(2E_f v_m ? + v_m E_f - E_f + E_m - E_m v_f -\ E_m v_f ?\right)\right)}{\left(E_f B_1 + E_m B_2\right)} \tag{S26}$$

Where

$$B_1 = 2\nu_m ? V_f - \nu_m + \nu_m V_f - \ 1 - V_f)$$

$$B_2 = 2\nu_f ? - V_f \nu_f - \ V_f \nu_f ? + V_f + \ \nu_f - 1)$$

$$E_T = 2\left(1 + v_{out-plane}\right) G_T \quad \text{(S27)}$$

$$v_{out-plane} = \frac{\left(K^{/} - mG_T\right)}{\left(K^{/} + mG_T\right)} \quad \text{(S28)}$$

Where

$$m = 1 + 4K^{/}\left(\frac{v_{in-plane}{}^{2}}{E_L}\right) \quad \text{(S29)}$$

$$K^{/} = \frac{K_m\left(K_f + G_m\right)V_m + K_f\left(K_m + G_m\right)V_f}{\left(K_f + G_m\right)V_m + \left(K_m + G_m\right)V_f} \quad \text{(S30)}$$

$$K_f = \frac{E_f}{2\left(1 + \nu_f\right)\left(1 - 2\nu_f\right)} \quad \text{(S31)}$$

$$K_m = \frac{E_m}{2\left(1 + \nu_m\right)\left(1 - 2\nu_m\right)} \quad \text{(S32)}$$

$$A\left(\frac{G_T}{G_{matrix}}\right) + 2B\left(\frac{G_T}{G_{matrix}}\right) + C = 0 \quad \text{(S33)}$$

$$A = 3V_f\left(1 - V_f\right) ? \left(\frac{G_f}{G_m} - \ \right)\left(\frac{G_f}{G_m} + \eta_{fiber}\right) + \left[\frac{G_f}{G_m}\eta_m + \eta_f\eta_m - \left(\frac{G_f}{G_m}\eta_m - \eta_f\right)V_f ?\right] \times \left[V_f\eta_m\left(\frac{G_f}{G_m} - \ \right) - \left(\frac{G_f}{G_m}\eta_m + 1\right)\right] \quad \text{(S34)}$$

$$B = -3V_f\left(1 - V_f\right) ? \left(\frac{G_f}{G_m} - \ \right)\left(\frac{G_f}{G_m} + \eta_f\right) + \left(\frac{1}{2}\right)\left[\eta_m\frac{G_f}{G_m} + \left(\frac{G_f}{G_m} - 1\right)\left(V_f + 1\right)\right] \times \left[\left(\eta_m - 1\right)\left(\frac{G_f}{G_m} + \eta_f\right) - 2\left(\frac{G_f}{G_m}\eta_m - \eta_f\right)V_f ?\right]$$
$$+ \left[\frac{V_f}{2}\left(\eta_m + 1\right)\left(\frac{G_f}{G_m} - 1\right)\right] \times \left[\left(\frac{G_f}{G_m} + \eta_f\right) + \left(\frac{G_f}{G_m}\eta_m - \eta_f\right)V_f ?\right] \quad \text{(S35)}$$

$$C = 3V_f\left(1 - V_f\right) ? \left(\frac{G_f}{G_m} - \ \right)\left(\frac{G_f}{G_m} + \eta_f\right) + \left[\eta_m\frac{G_f}{G_m} + \left(\frac{G_f}{G_m} - 1\right)V_f + 1\right] \times \left[\left(\frac{G_f}{G_m} + \eta_f\right) + \left(\frac{G_f}{G_m}\eta_m - \eta_f\right)V_f ?\right] \quad \text{(S36)}$$

Where,

$V_{out\text{-}plane}$: out-of-plane Poisson’s ratio

m : scaling factor

K: stiffness parameter

Composite Density

$$\rho_c = \rho_f V_f + \rho_m V_m \tag{S37}$$

Where,

ρ_c : Density of composite

ρ_f : Density of the fibre

ρ_m : Density of the matrix

V_f : Volume fraction of the fibre in the composite

V_m : Volume fraction of the matrix in the composite

Comparative Study

$$A_E = \frac{E_L}{E_T} \tag{S38}$$

$$\eta E_L = \frac{E_{L(comp)} - E_m V_m}{E_f V_f} \tag{S39}$$

$$HEI = \frac{E_{L(hyb)} - \sum w_i E_{L(i)}}{\sum w_i E_{L(i)}} \times 100\% \tag{S40}$$

$$Specific\ stiffness = \frac{E_L}{\rho} \tag{S41}$$

Where,

A_E : Anisotropy ratio of the composite

ηE_L : Reinforcement efficiency of the composite

HEI : Hybrid effect index of the composite

w_i : Relative volume fraction of each fibre in the composite
